# National Black HIV/AIDS Awareness Day — February 7, 2015

**Published:** 2015-02-06

February 7 is National Black HIV/AIDS Awareness Day, an observance intended to raise awareness of human immunodeficiency virus/acquired immunodeficiency syndrome (HIV/AIDS) and encourage action, such as HIV testing, to reduce the disproportionate impact of HIV/AIDS on blacks or African Americans in the United States. Two of the three goals of the National HIV/AIDS Strategy are to reduce new HIV infections and HIV disparities (1).

Compared with other races and ethnicities, blacks had the highest HIV incidence in 2010, with an estimated rate of 68.9 per 100,000 population, which was nearly eight times the estimated rate of 8.7 among whites (2). By the end of 2011, an estimated 491,100 of the estimated 1.2 million persons living with HIV in the United States were blacks, accounting for the highest percentage (41%) of persons living with HIV, followed by whites (34%) and Hispanics/Latinos (20%) (3). Among blacks living with HIV in 2011, 85% had their infection diagnosed, 40% were engaged in care, 36% were prescribed antiretroviral therapy, and 28% were virally suppressed (4).

Information about National Black HIV/AIDS Awareness Day is available at http://www.cdc.gov/features/blackhivaidsawareness. Information about blacks and HIV/AIDS is available at http://www.cdc.gov/hiv/risk/racialethnic/aa/index.html.
